# The application of antimicrobials in VAP patients requiring ECMO supportive treatment

**DOI:** 10.3389/fphar.2022.918175

**Published:** 2022-09-23

**Authors:** Dongna Zou, Mei Ji, Tingting Du, Qian Wang, Haiwen Zhang, Hengcai Yu, Ning Hou

**Affiliations:** ^1^ Department of Pharmacy, Shandong Provincial Hospital Affiliated to Shandong First Medical University, Jinan, China; ^2^ Department of Stomatology, Shandong Provincial Hospital Affiliated to Shandong First Medical University, Jinan, China; ^3^ Department of Pharmacy, Jinan Second People's Hospital, Jinan, China

**Keywords:** ECMO, VAP, antimicrobials, dosing regimen, PK

## 1 Introduction

Extracorporeal membrane oxygenation (ECMO), as we all know, is a therapeutic modality that provides life support for critically ill patients with severe respiratory or heart failure as an extracorporeal life support, gaining valuable time for the recovery of their cardiopulmonary function and subsequent treatment (Chen H et al., 2014; Chaves RCF et al., 2019; Combes A et al., 2018).

Although it is life-saving for patients with cardiorespiratory failure, being an invasive treatment, it invariably poses new care issues, such as an extra risk factor for concomitant infections (Chaves RCF et al., 2019).

Factors such as systemic inflammatory response, changes in the body fluid status, serum albumin concentration, changes in liver and kidney functions, and usage of continuous renal replacement therapy will all alter the pharmacokinetics (PK) of medications to variable degrees in severe pathophysiological situations (Fleuren LM et al., 2019).

Furthermore, the ECMO circuit will cause complicated alterations in medication PK (Dzierba AL et al., 2017). A lot of studies have demonstrated that ECMO affects drug PK by adsorbing, increasing the apparent volume of distribution (Vd), and decreasing the clearance (CL) (Wildschut et al., 2010; Shekar et al., 2012; Lemaitre et al., 2015; Mehta NM et al., 2007; Shekar et al., 2015). So if the antibiotic dosage is inappropriate due to the influence of multiple factors, it may result in treatment failure due to insufficient dosage, drug resistance of pathogenic bacteria, or even endangering the patient’s life, and empirically increasing the drug dosage may result in drug accumulation or drug toxicity.

Therefore, understanding the PK changes of antibiotics in critically ill patients undergoing ECMO supportive therapy is vital for the selection of antibiotics and the design of tailored treatment regimens for this patient population, such as dose modification.

At the same time, since patients receiving ECMO must use a ventilator to simulate breathing in order to maintain lung function, long-term use of a ventilator may damage the normal respiratory mucosal barrier, reduce ciliary movement and defense function, and it may also result in the decrease of resident bacteria in the oral cavity, increased bacterial reproduction, aspiration and accumulation of respiratory secretions, and incomplete disinfection of the ventila.

Furthermore, because pulmonary infection is the most common site of nosocomial infection under ECMO support treatment, the incidence rate is much higher than that of other sites, and the pathogen resistance rate and minimum inhibitory concentration (MIC) value of ventilator-associated pneumonia (VAP) patients may differ from those of common pneumonia.

As a result, the purpose of this article is to offer patients with suitable dose regimens by describing the antibiotic therapy of patients with VAP who receive ECMO treatment.

### 2 Epidemiological features of ventilator-associated pneumonia patients undergoing extracorporeal membrane oxygenation treatment

According to a 2013 research, the most frequent isolate among patients with VAP getting ECMO treatment was Enterobacteriaceae (n = 13; 56%). Other Gram-negative bacilli and methicillin-resistant *Staphylococcus aureus* (MRSA) were responsible for six (26%) and five (22%) of the VAP episodes, respectively (Aubron C et al., 2013). Schmidt et al. (2012) discovered that *Pseudomonas aeruginosa* was the most often cultivated bacteria from lung samples (26%) after reviewing a large series of 220 patients who got venous-arterial ECMO (VA-ECMO). Another study looked at 152 patients who had ECMO assistance for more than 48 h and discovered that the primary infections were due to *Pseudomonas aeruginosa* (51%) and Enterobacteriaceae (35%) (Bouglé et al., 2018). The percentage was similar to that reported after cardiac surgery, and the bacterial translocation was thought to be related to a circulatory collapse (Allou N et al., 2013).

### 3 Possible extracorporeal membrane oxygenation mechanisms affecting drug pharmacokinetics

Lipophilicity is one of the most critical parameters influencing medication PK. The higher the lipophilicity, the more the organic compounds are soluble in the organic phase (Testa, B. et al., 2000).

Because the ECMO circuit is largely made up of organic components, such as polyvinylchloride tubing and hollow fiber oxygenators, these materials will also impact drug PK, and medicines with higher lipophilicity are more likely to be absorbed (Preston et al., 2007; Preston et al., 2010; Shekar et al., 2012; Ghazi Suliman et al., 2017).

Aside from lipophilicity, the drug–protein binding rate is a key component in influencing the degree of drug adsorption by the ECMO circuit. Two *in vitro* ECMO tests revealed that medicines with a high protein-binding rate, such as ceftriaxone and caspofungin, were greatly reduced in the ECMO circuit (Wildschut et al., 2010; Shekar et al., 2015).

In addition to changing the Vd of medications by adsorption in the circuit outlined previously, ECMO may raise the Vd of pharmaceuticals through hemodilution and hemodynamic changes. Perfusion of the ECMO circuit, blood product infusion, and fluid resuscitation all raise the patient’s circulating blood volume, generating hemodilution and raising the drug’s Vd. During ECMO, inflammatory reactions can cause capillary leakage and edema, resulting in elevated Vd. Non-pulsatile blood flow in VA-ECMO may cause renin–angiotensin system upregulation, leading to reduced urine production and salt excretion, further raising Vd (Many et al., 1967).

Drug clearance changes in ECMO-supported patients have been studied. Significant differences in decreased clearance (CL) of gentamicin and ticarcillin/clavulanate, and increased CL of caspofungin and micafungin were reported in pediatric patients on ECMO compared to non-ECMO controls (Cohen et al., 1990; Lindsay et al., 1996; Spriet et al., 2009; Autmizguine et al., 2016).

## 4 PK alterations in routinely used antibiotics during extracorporeal membrane oxygenation

Because this article primarily addresses the therapy of antibiotics for patients with VAP who were on ECMO, the application of frequently used antibiotics during ECMO, including the dose and mode of administration, is listed.

### 4.1 Linezolid

De Rosa et al. (2013) discovered that when linezolid was used as an intermittent infusion of 600 mg every 12 h in three ECMO patients, plasma pharmacodynamic targets were easily achieved only when the *S. aureus* MIC is ≤1 mg/L, and prolonged or continuous infusion, as well as increased dosage, may be required when *S. aureus* with MICs >1 mg/L. Another study by Kühn et al. (2020) found that even with a greater dosage of 1,800 mg/d of linezolid for continuous infusion, ideal target blood concentrations were not achieved in 35% of ECMO patients. Nikolos et al. (2020) came to the same result that linezolid did not attain target plasma concentrations at a dosage of 600 mg every 8 h in a patient receiving simultaneous venovenous ECMO assistance.

As a result, we recommend increasing the dosage of linezolid and doing therapeutic drug monitoring (TDM) whenever possible, especially in the case of patients with MICs greater than 1 mg/L.

### 4.2 Vancomycin

Based on current research, different *in vitro* studies have not reached a consistent conclusion on how ECMO affected vancomycin PK, both across adults and between adults and children (Amaker et al., 1996; Buck, 1998; Shekar et al., 2012; Lemaitre et al., 2015; Raffaeli et al., 2020).

Donadello et al. (2014) and Moore et al. (2016) discovered that the PK was comparable between ECMO and non-ECMO patients, and the standard dose was appropriate.

In contrast to this viewpoint, another study found that vancomycin levels in the MiniLung petite dropped by 38% (Raffaeli et al., 2020). According to further adult data from Kim SJ, vancomycin was frequently underdosed, and serum concentrations were subtherapeutic due to a hemodilutional impact (Park et al., 2015).

It is worth noting that the PK of vancomycin demonstrated that CL reduced considerably in neonates receiving ECMO compared to non-ECMO controls (Buck, 1998; An et al., 2020). Several previous investigations found that vancomycin had higher Vd, CL, and half-life in pediatric ECMO patients. The mean half-life, for example, varied from 6.53 to 16.9 h, implying a dosage interval of 18–24 h (Hoie et al., 1990; Amaker et al., 1996; Buck, 1998; Mulla and Pooboni, 2005).

Another research found that an initial dosage frequency of every 12 h to deliver a dose of 15 mg/kg/dose was a viable choice in pediatric ECMO patients with normal renal function at baseline (Lonabaugh et al., 2017).

As stated previously, there is no clear consensus on whether the standard dosage of vancomycin should be modified during ECMO. TDM is still indicated for the safe and effective management of ECMO patients undergoing vancomycin therapy.

### 4.3 Teicoplanin

Chen et al. (2020) showed that a loading dose of four doses of teicoplanin at a dose of 12 mg/kg/dose delivered within the first 72 h may establish an adequate therapeutic Ctrough of teicoplanin in patients receiving VA-ECMO.

For mild–moderate infections, Wi et al. (2017) suggested a loading dose (LD) of 600 mg and a maintenance dose (MD) of 400 mg for ECMO patients not receiving continuous renal replacement therapy (CRRT) and an LD of 800 mg and an MD of 600 mg for those receiving CRRT, and for severe infections, the ideal dosage was 1,000 mg with an MD of 800 mg for ECMO patients who were not getting CRRT and 1,200 mg with an MD of 1,000 mg for those who were receiving CRRT.

According to studies, greater dosages were required to obtain rapid and adequate teicoplanin exposure.

### 4.4 Gentamicin

Current gentamicin PK investigations in ECMO patients have mostly focused on newborns and children. According to the literature, the PK of gentamicin in patients receiving ECMO differed inconsiderably in neonates compared to those who did not receive ECMO (Cohen et al., 1990; Munzenberger and Massoud, 1991; Bhatt-Mehta et al., 1992).

However, new research based on a population PK analysis of gentamicin revealed a lower estimated CL in children and adolescents receiving ECMO and a longer dose interval and an empiric dosing schedule of 4–5 mg/kg every 24 h for neonates and infants (Moffett et al., 2018).

As a result, we believe that the dose changes, and TDM will be required on a regular basis.

### 4.5 Piperacillin-tazobactam

A model developed by Hahn et al. (2021) demonstrated that CL decreased in patients who received ECMO compared to non-ECMO controls, and continuous infusion in critically ill patients with creatinine clearance (CrCL) of ≥60 ml/min was suggested, with at least 12,000, 16,000, and 20,000 mg/day required for CrCL of <40, 40–60, and 60–90 ml/min, respectively. In patients with CrCL of ≥90 ml/min, a continuous infusion of at least 24,000 mg/day was required.

Fillâtre P et al., on the other hand, showed that ECMO support had no effect on piperacillin exposure, while ICU patients with sepsis were commonly underexposed to piperacillin, indicating that TDM should be highly advised for severe infections (Fillâtre P et al.).

### 4.6 Meropenem

Many studies have shown that there was no difference in CL for meropenem between ECMO and non-ECMO patients; hence, there was no need to consider ECMO or not when meropenem dosage optimization was used (Donadello et al., 2015; Cies et al., 2016; Andresen et al., 2018; Lee D.-H. et al., 2021; Gijsen et al., 2021; Wang et al., 2021).

However, Abdul-Aziz MH et al. proposed that the beta-lactam dose in ECMO patients should typically correspond to the recommended dosing techniques for critically ill non-ECMO patients (Abdul-Aziz and Roberts, 2020).

A higher dose with longer infusion was advocated for patients with elevated CrCL, or if more aggressive, objectives such as 100% fT>MIC or 100% fT > 4xMIC were used (Zylbersztajn et al., 2021). Korean researchers voiced similar thoughts, suggesting a 3-h infusion or a high-dosage regimen of 2,000 mg/8 h (Lee JH. et al., 2021).

Given the loss of meropenem in the ECMO circuit (Shekar et al., 2012) and the likelihood of elevated MIC values for pathogenic bacteria, higher-than-usual dose recommendations may be necessary, and TDM must be considered.

### 4.7 Imipenem

Like meropenem, imipenem is a carbapenem; however, there are peculiarities. Most research on imipenem shows that ECMO patients should increase their dose.

The prescribed dosages of imipenem (1,000 mg three times a day) did not attain the PK objectives for the majority of patients, according to a study (Bouglé et al., 2019). Because blood concentrations in ECMO patients were found to be lower than those in non-ECMO patients, the dose should be raised to 750 mg every 6 h (Chen GJ. et al., 2020).

Unlike Chen W, Jaruratanasirikul et al. (2021) discovered that the ECMO circuit had minimal effect on improving the PK alterations of imipenem that had already happened in critically ill patients. However, they stated that a large dose of imipenem (such as a 4-h infusion with a dose of 1,000 mg q6h) may be necessary to achieve the PK/pharmacodynamic (PD) objectives against less sensitive bacteria with MICs of 4 mg/L (Jaruratanasirikul et al., 2021). This viewpoint is congruent with the study published in 2020 by Jaruratanasirikul et al. (2020).

According to a 2019 study, pathophysiological changes in critically ill patients with severe infections receiving ECMO had a greater impact on altered PK patterns of imipenem than those not receiving ECMO; thus, a high dose of imipenem (1,000 mg every 6 h) may be required to maintain adequate drug concentrations to achieve the PK/PD targets (Jaruratanasirikul et al., 2019).

Welsch et al. (2015) discovered that an increased dose schedule (4,000 mg/24 h) was more likely to optimize medication exposure in lung transplantation patients on ECMO.

As a result of the aforementioned analysis of the literature, we proposed a greater dose of imipenem in ECMO patients than in non-ECMO patients, and the recommended dosage is 750–1000 mg, q 6 h.

### 4.8 Ciprofloxacin

Sinnah et al. (2017) came to the conclusion that in the *ex vivo* model of ECMO circuits, the median concentrations of ciprofloxacin at baseline and 24 h after ciprofloxacin infusion were similar, so ciprofloxacin dosing in ECMO patients should remain in line with the recommended dosing strategies for critically ill patients not receiving ECMO, which was consistent with the previous literature, and it can be seen that there was no significant loss of ciprofloxacin (Shekar et al., 2015).

According to the findings of most recent research studies, there is no need to adjust the standard dose of ciprofloxacin for ECMO patients, although we still advocate monitoring ciprofloxacin blood concentrations to assure therapeutic effect if situations permit.

### 4.9 Tigecycline

Due to the prevalence of multidrug-resistant pathogens and the lack of novel antibacterial drugs, tigecycline was expanded to treat additional serious infections using a high-dose tigecycline regimen. According to the studies, the efficacy of off-label high dosage tigecycline in VAP was required (200 mg followed by 100 mg every 12 h) (G. Dimopoulos et al., 2022; R. Zaragoza et al., 2020).

Zhang Y’s *ex vivo* investigation was meant to investigate the sequestration of various antibacterials, including tigecycline, in ECMO circuits; no tigecycline loss was determined (Zhang et al., 2021), which was compatible with the literature published in 2012 (Veinstein et al., 2012).

As a result, we advise a high-dose tigecycline regimen for patients with VAP who were on ECMO, such as 200 mg followed by 100 mg every 12 h, although more study is needed to corroborate this.

## 5 Conclusion

Although we discuss the epidemiological features of VAP patients receiving ECMO therapy, putative ECMO processes impacting medication pharmacokinetics and the PK alterations of frequently used antibiotics during ECMO, including the dose and mode of administration (Table 1). However, the current evidence limitations, such as the small sample size, and the patient’s own conditions, such as pathophysiology, may affect the drug’s PK changes; in particular, it is the patient’s own factors that can lead to an inadequate concentration of antimicrobials, such as leaky capillaries, hypoalbuminemia, augmented renal clearance, and impaired tissue penetration in septic patients (Roberts and Lipman, 2009; Ulldemolins et al., 2011; Shekar et al., 2013; Bilbao-Meseguer et al., 2018). Further research, including large-scale studies, will be required in the future to give appropriate dose recommendations for physicians and patients. When conditions allow, the dosing regimen can be adjusted with the use of TDM. However, our findings should serve as a reminder to doctors and clinical pharmacists to pay more attention to antibiotic administration for patients with VAP who are on ECMO. In the absence of further research studies, our findings can serve as a guide for doctors when prescribing popular antibiotics for VAP.

**TABLE 1:** Administration recommendations of antimicrobials in VAP patients requiring ECMO supportive treatment.

Antimicrobial	Applicable population	Medication recommendation	Literature
Linezolid	*S. aureus* with MICs≤1 mg/L	Intermittent infusion of 600 mg every 12 h	De Rosa et al. (2013)
	*S. aureus* with MICs >1 mg/L	Prolonged or continuous infusion of 600 mg every 12 h	
	−	High dose	Kühn et al., 2020; Nikolos et al., 2020
Vancomycin	−	Currently unclear	
Teicoplanin	−	High dose: LD 12 mg/kg/dose, 4 doses	Chen et al. (2020)
	Without CRRT, mild to moderate infections	LD 600 mg and MD 400 mg	Wi et al. (2017)
	With CRRT, mild to moderate infections	LD 800 mg and MD 600 mg	
	Without CRRT, severe infections	LD 1,000 mg and MD 800 mg	
	With CRRT, severe infections	LD 1,200 mg and MD 1,000 mg	
Gentamicin	−	Currently unclear	
Piperacillin-tazobactam	CrCL: < 40 ml/min	12 g/day	Hahn et al. (2021)
	CrCL: 40–60 ml/min	16 g/day	
	CrCL: 60–90 ml/min	20 g/day, continuous infusion	
	CrCL: ≥90 ml/min	24g/day at least, continuous infusion	
	−	Conventional dose, but TDM should be strongly recommended for severe infections	Fillâtre et al. (2021)
Meropenem	−	Currently unclear	
Imipenem	−	High dose: 750–1000 mg, q6 h	Chen et al., 2020a; Jaruratanasirikul et al., 2019; Jaruratanasirikul et al., 2020; Jaruratanasirikul et al., 2021; Welsch et al., 2015
Ciprofloxacin	−	Conventional dose	Shekar et al., 2015; Sinnah et al., 2017
Tigecycline	−	Conventional dose in VAP	Our team

MIC, minimum inhibitory concentration; LD, loading dose; MD, maintenance dose; CRRT, continuous renal replacement therapy; CrCL, creatinine clearance; VAP, ventilator-associated pneumonia.
